# *ScChi*, Encoding an Acidic Class III Chitinase of Sugarcane, Confers Positive Responses to Biotic and Abiotic Stresses in Sugarcane

**DOI:** 10.3390/ijms15022738

**Published:** 2014-02-18

**Authors:** Yachun Su, Liping Xu, Zhiwei Fu, Yuting Yang, Jinlong Guo, Shanshan Wang, Youxiong Que

**Affiliations:** Key Laboratory of Sugarcane Biology and Genetic Breeding, Ministry of Agriculture, Fujian Agriculture and Forestry University, Fuzhou 350002, China; E-Mails: syc2009mail@163.com (Y.S.); fuzhiwei1991@126.com (Z.F.); yytjiayou@126.com (Y.Y.); jl.guo@163.com (J.G.); wangshan614@126.com (S.W.)

**Keywords:** chitinase, *Saccharum officinarum*, *Sporisorium scitamineum*, subcellular localization, gene expression, abiotic stress

## Abstract

Chitinases (EC 3.2.2.14), expressed during the plant-pathogen interaction, are associated with plant defense against pathogens. In the present study, a positive correlation between chitinase activity and sugarcane smut resistance was found. *ScChi* (GenBank accession no. KF664180), a Class III chitinase gene, encoded a 31.37 kDa polypeptide, was cloned and identified. Subcellular localization revealed ScChi targeting to the nucleus, cytoplasm and the plasma membrane. Real-time quantitative PCR (RT-qPCR) results showed that *ScChi* was highly expressed in leaf and stem epidermal tissues. The *ScChi* transcript was both higher and maintained longer in the resistance cultivar during challenge with *Sporisorium scitamineum*. The *ScChi* also showed an obvious induction of transcription after treatment with SA (salicylic acid), H_2_O_2_, MeJA (methyl jasmonate), ABA (abscisic acid), NaCl, CuCl_2_, PEG (polyethylene glycol) and low temperature (4 °C). The expression levels of *ScChi* and six immunity associated marker genes were upregulated by the transient overexpression of *ScChi*. Besides, histochemical assay of *Nicotiana benthamiana* leaves overexpressing pCAMBIA 1301-*ScChi* exhibited deep DAB (3,3′-diaminobenzidinesolution) staining color and high conductivity, indicating the high level of H_2_O_2_ accumulation. These results suggest a close relationship between the expression of *ScChi* and plant immunity. In conclusion, the positive responses of *ScChi* to the biotic and abiotic stimuli reveal that this gene is a stress-related gene of sugarcane.

## 1. Introduction

Plants are vulnerable to external environmental stimuli, as they lack an effective immune system. When under attack from pathogen infection, plants activate a number of defense responses to cope with infection and to protect themselves [1]. Chitinases (EC 3.2.2.14), which can catalyze poly chitin, are present in the cell walls of most fungi and homologues in plant typical pathogenesis-related (PR) proteins. Previous research has revealed that they play an important role in plant defense mechanisms [2,3]. Chitinases isolated from both monocot and dicot plants have been shown to inhibit the growth of chitin-containing fungi, both *in vitro* [4,5] and *in vivo* [6,7]. Plant chitinases also have resistance characteristics implicated in plant defense to different kinds of pathogens, including bacteria, viruses and other abiotic stresses [8]. Chitinase isozymes are a diverse group of enzymes that differ in enzymatic activities, primary sequence, isoelectric point (pI) and cellular localization [9]. Chitinase genes have been grouped into seven classes (Class I–VII) belonging to the glycoside hydrolase families 18 and 19 [2,10].

Sugarcane smut, caused by the fungus *Sporisorium scitamineum* (*S. scitamineum*), is a worldwide disease and causes serious losses in sugarcane yield and sucrose content [11–13]. Several methods, such as effective agronomic practices and chemical control, have been recommended for the control of smut infection. However, only the use of resistant cultivars has proven to be practical and economical [14]. With the development of biotechnological approaches, crop protection by engineering genetic disease resistance is becoming a promising tool to eliminate the disadvantages of traditional methods [10]. As reported, transgenic plants overexpressing PR proteins could enhance resistance against fungal diseases (e.g., the isolectin I purified from the transgenic *Nicotiana tabacum*, which transformed an isolectin I gene from *Urtica dioica*, showed *in vitro* antifungal activities on germinated spores of the fungi, *Botrytis cinerea*, *Trichoderma viride* and *Colletotrichum lindemuthianum*) [15]. However, isolating and characterizing the gene that encodes a broad-spectrum antifungal protein is a prerequisite for obtaining promising transgenic plants resistant to fungal pathogens.

Plant chitinases have been the most extensively studied and applied PR proteins. To date, a wide variety of plant chitinases have been identified from several plant species, including *Arabidopsis thaliana*, *Oryza sativa*, *Nicotiana tabacum*, *Triticum aestivum* and *Zea mays* [7,10,16]. Class I chitinase from *Hordeum vulgare* and Class VII chitinase from *T. aestivum* have been reported to demonstrate antifungal activities in recent studies [10,17]. Transgenic banana (*Musa acuminata*) integrating rice chitinase gene exhibited resistance to black leaf streak disease caused by the pathogenic fungus, *Mycosphaerella fijiensis* [16]. In sugarcane, only one full-length chitinase gene, *ScChiB1* (EU914815.1), belonging to the Class IV of family 19 glycosyl hydrolases, has been amplified from the red rot resistant cultivar, Co93009 [18]. The relative expression of this chitinase gene in both the red rot resistant (Co 93009) and susceptible (CoC 671) cultivars by real-time quantitative PCR (RT-qPCR) showed a high level of expression only in the resistant cultivar during interaction with *Colletotrichum falcatum*. In addition, the chitinases from the leaves of the chewing cane (*Saccharum officinarum*) cultivar Fuan were upregulated after *C. falcatum* infection, and an increasing expression pattern of a partial chitinase gene has also been validated by RT-qPCR at 24, 48 and 72 h of interaction [19].

In the present study, an activity assay of chitinase enzymes was firstly conducted on sugarcane cultivars (resistant and susceptible genotypes) in response to *S. scitamineum* pathogen. Secondly, a full-length chitinase gene, *ScChi* (GenBank accession no. KF664180) from sugarcane challenged by *S. scitamineum* was isolated and characterized. Finally, its expression in *Escherichia coli* (*E. coli*) cells, probable localization, relative mRNA expression in sugarcane tissues and various expression profiles under different biotic/abiotic stresses, as well as transient expression in *Nicotiana benthamiana* were investigated. This study aims to obtain better knowledge of sugarcane chitinases and the function of their encoding genes.

## 2. Results and Discussion

### 2.1. Enzyme Activity of Chitinase

**Figure 1.**
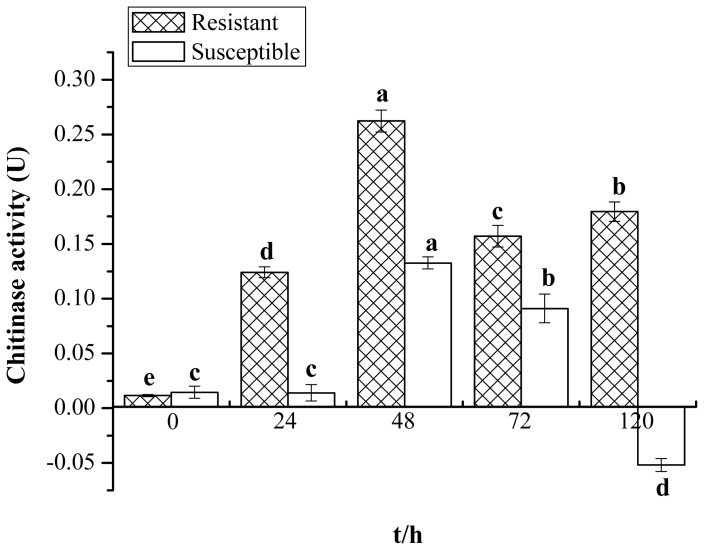
The chitinase activity in smut-resistant (Yacheng05–179) and smut-susceptible (Liucheng03–182) sugarcane cultivars inoculated with *Sporisorium scitamineum*. All data points (with the deduction of their mocks) are the means ± SE (*n* = 3). Different lowercase letters indicate a significant difference, as determined by the least-significant difference test (*p*-value < 0.05). U, unit of chitinase activity.

A biochemical method for the determination of enzyme activity was applied to analyze the change in chitinase activity in Yacheng05–179 (resistant) and Liucheng03–182 (susceptible) harvested at different time points after smut infection. Figure 1 shows that the elevated activity of chitinase in Yacheng05–179 can be observed at an early stage of 24 h compared to the control. Activity remains at a higher level at all later time points. The maximum level of activity was observed at 48 h; then, the accumulated level slightly decreases at 72 h before increasing to 0.1795 U (unit of chitinase activity (U)) at 120 h. The activity of chitinase in Liucheng03–182 remained unchanged at the first 24 h and reached the peak value of 0.1325 U at 48 h, then decreased at 72 h before being much reduced at 120 h. It is interesting that chitinase activity in the resistant cultivar is always higher than that of the mock and the susceptible cultivar challenged by smut pathogen, suggesting that a positive correlation may exist between sugarcane chitinase activity and smut resistance.

### 2.2. Cloning and Sequence Analysis of the Chitinase Gene, *ScChi*

**Figure 2.**
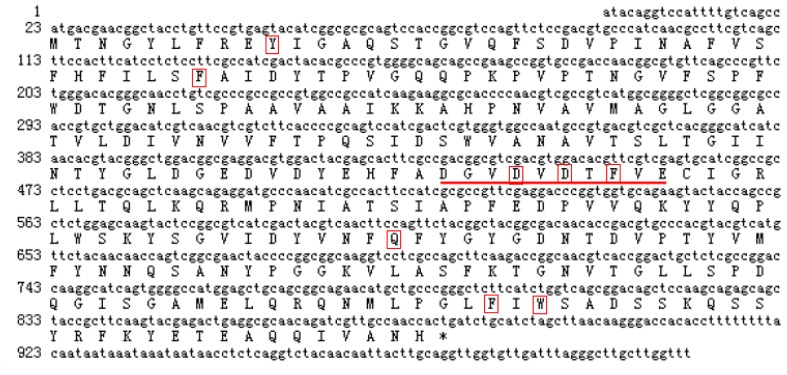
Nucleotide acid sequences obtained by real-time (RT)-PCR and the deduced amino acid sequences of *ScChi*. The deduced amino acid sequences are shown as a one-letter code under the cDNA sequences. The underline indicates the catalytic domains (DXXDXDXXXE) of the glycosyl hydrolase family 18. The eight red squares of Tyr_10_ (Y_10_), Phe_37_ (F_37_), Asp_140_ (D_140_), Asp_142_ (D_142_), Phe_144_ (F_144_), Gln_195_ (Q_195_), Phe_259_ (F_259_) and Trp_261_ (W_261_) show putative carbohydrate binding sites.

A sugarcane chitinase-like gene, *ScChi* (GenBank accession no. KF664180), was isolated from Yacheng05–179 post-48 h *S. scitamineum* inoculation by RT-PCR-based cloning. *ScChi* had a full length of 1001 bp, with an open reading frame (ORF) of 861 bp (encoding a polypeptide of 286 amino acids) (Figure 2). The predicted protein had a molecular weight of 31.37 kDa with a pI of 4.69. MotifScan software (SIB, Geneva, Switzerland) showed that ScChi contained a glycosyl hydrolase family 18 (Pfam) domain from position 32 to 266 aa, which suggested that sugarcane *ScChi* encoded a putative chitinase. The NetNGlyc online program analysis predicted three putative *N*-glycosylation sites (at position 65, 214 and 232 aa) with probabilities of 20.18%, 49.02% and 68.47%, respectively. There was no signal peptide and transmembrane helix domain in ScChi by SignalP (CBS, New York, NY, USA) and TMHMM Server v. 2.0 programs (CBS, New York, NY, USA), which suggested that ScChi was not a secretory protein. ScChi contained the active site of the catalytic residues, Asp_137_ (D_137_), Asp_140_ (D_140_), Asp_142_ (D_142_) and Glu_146_ (E_146_) (Figure 2).

**Figure 3.**
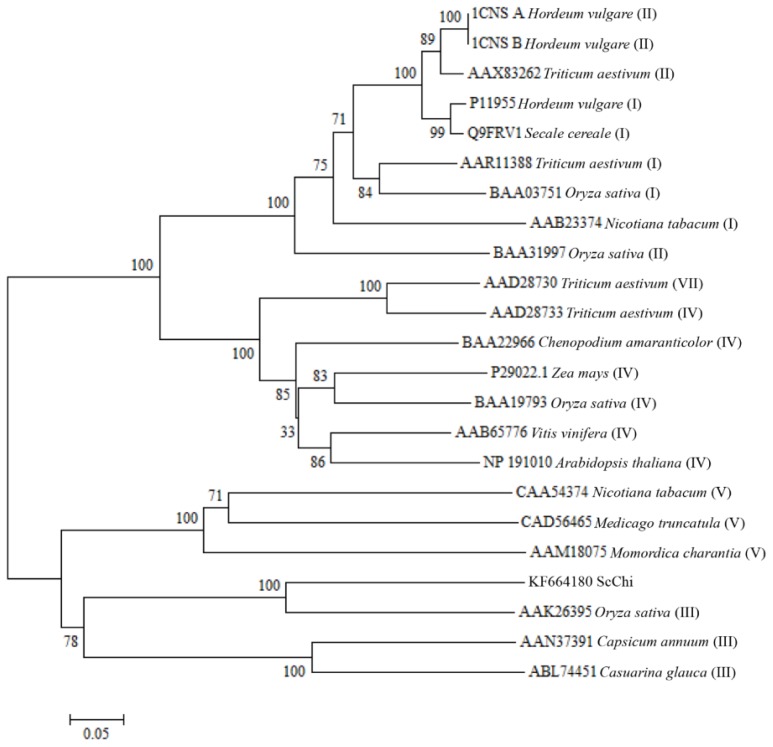
Phylogenetic analysis of deduced amino acid sequence from ScChi (KF664180) and other plant chitinases. The neighbor-joining method was used. Plant chitinase classes are represented in parentheses.

**Figure 4.**
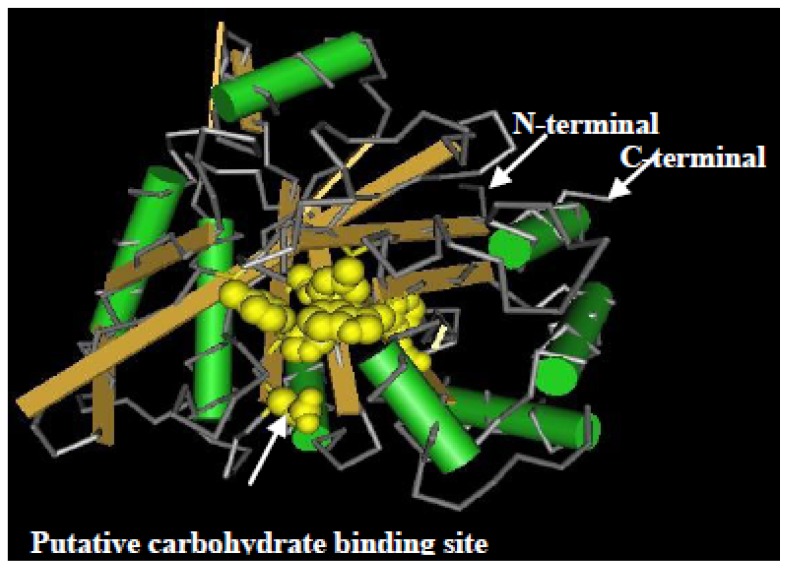
Three-dimensional structural analysis of the GH18_narbonin (cd06544) domain, which was similar to ScChi by Blastp. The presence of putative carbohydrate binding sites, which was highly homologous to ScChi, is shown in the 3D structure of GH18_narbonin by arrows.

Basic Local Alignment Search Tool (BLAST) search of the amino acid sequences indicated that ScChi shared high sequence similarity with diverse plant chitinases in the GenBank database. The predicted amino acid sequence of *ScChi* showed 84.27% identity with that of *Z. mays* (AFW72831.1), 75.78% with that of *Aegilops tauschii* (EMT04102.1), 73.61% with that of *O. sativa* (NP_001064607.1) and 73.45% with that of *Triticum urartu* (EMS51632.1). The phylogenetic tree showed that ScChi was closely related to *O. sativa* chitinase (III) (AAK26395) (Figure 3). Three-dimensional structures (3D) of the GH18_narbonin (cd06544) similar to ScChi domain were found by Blastp in NCBI (http://www.ncbi.nlm.nih.gov/Structure/cdd/wrpsb.cgi?RID=FSWDHR9T014&mode=all) (Figure 4). The structure of GH18_narbonin showed the presence of a putative carbohydrate binding site, which was highly homologous to ScChi (the residues were Tyr_10_ (Y_10_), Phe_37_ (F_37_), Asp_140_ (D_140_), Asp_142_ (D_142_), Phe_144_ (F_144_), Gln_195_ (Q_195_), Phe_259_ (F_259_) and Trp_261_ (W_261_)) (Figure 2). These combined data suggested that ScChi belonged to an acid Class III chitinase, the member of family 18 glycosyl hydrolase.

### 2.3. Subcellular Localization of *ScChi*

**Figure 5.**
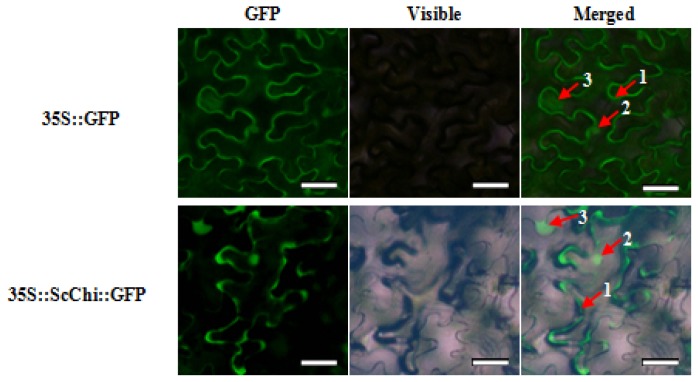
The subcellular localization analysis of ScChi in *Nicotiana benthamiana* leaves 48 h after infiltration. The epidermal cells were used for taking images of green fluorescence, visible light and merged light. Read Arrows 1, 2 and 3 indicated plasma membrane, nucleus and cytoplasm, respectively. Bar = 50 μm.

*ScChi* was cloned into the plant expression vector of pCAMBIA 2300 containing the CaMV 35S promoter and the *GFP* reporter gene. *Agrobacterium*-mediated transformation was then performed to identify the transient expression of the target gene and *GFP* in *N. benthamiana*. Infiltrated leaves incubated for 48 h at 24 °C were collected, and the green fluorescence was monitored under a fluorescence microscope. As illustrated in Figure 5, compared with the mock, ScChi::GFP fusion protein was observed in the nucleus, cytoplasm and potentially associated with the plasma membrane.

### 2.4. Prokaryotic Expression of *ScChi* in *E. coli* Rosetta Cells

**Figure 6.**
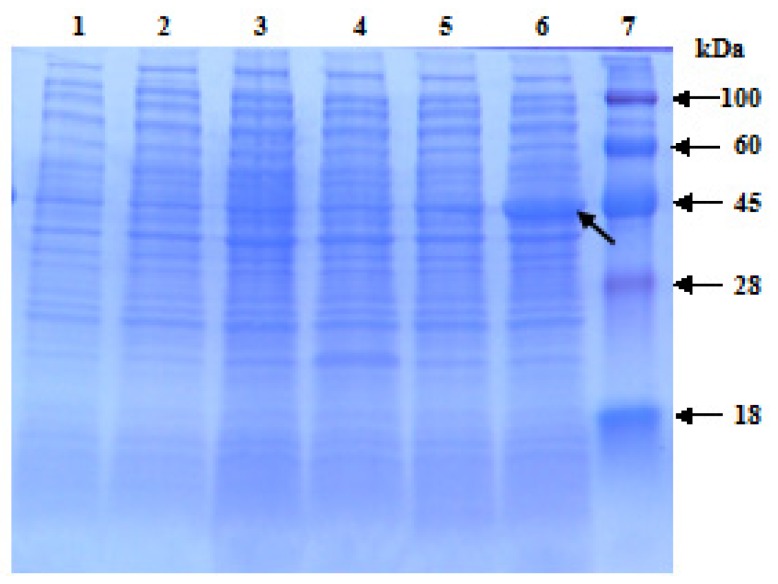
The prokaryotic expression of pET 32a-*ScChi* in *Escherichia coli* Rosetta cells. 1, blank (*E. coli* Rosetta cells) without induction; 2, blank induction for 1 h; 3, control (*E. coli* Rosetta cells containing pET 32a vector) without induction; 4, control induction for 1 h; 5, pET 32a-*ScChi* without induction; 6, pET 32a-*ScChi* induction for 1 h; 7, protein marker; the induced protein is shown by the arrow.

The ORF (861 bp) of *ScChi* was subcloned into expression vector pET 32a (+) under the control of an inducible promoter and transformed into *E. coli* Rosetta cells. Sodium dodecyl sulfate-polyacrylamide gel electrophoresis (SDS-PAGE) analysis showed the accumulation of the recombinant protein in LB medium in response to 1.0 mM isopropyl β-d-thiogalactoside (IPTG) induction at 28 °C (Figure 6). At the presence of 6× His-tag, the target protein showed higher molecular weight than the estimated one of 31.37 kDa.

### 2.5. Overexpression of *ScChi* in *E. coli* Enhances Cell Growth under Abiotic Stresses

**Figure 7.**
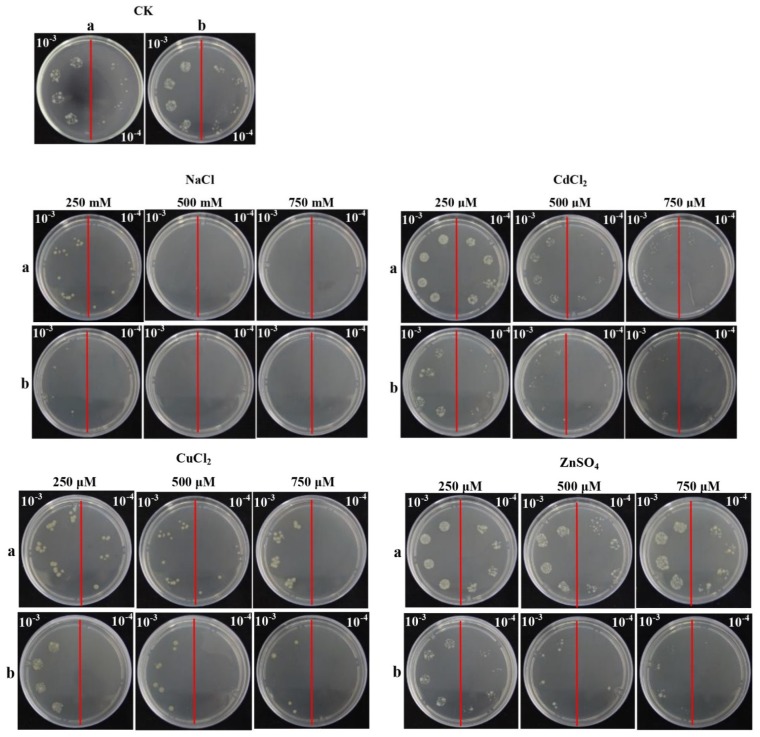
Spot assays of Rosetta + pET 32a-*ScChi* (**a**) and Rosetta + pET 32a (control) (**b**) on Luria-Bertani (LB) plates with NaCl, CuCl_2_, CdCl_2_ and ZnSO_4_. Isopropyl β-d-thiogalactoside (IPTG) was added to the cultures of Rosetta + pET 32a-*ScChi* and Rosetta + pET 32a to induce the expression of recombinant protein. The cultures were adjusted to OD_600_ = 0.6. Ten microliters from 10^−3^ (left side of the red line on the plate) to 10^−4^ (right side of the red line on the plate) dilutions were spotted onto LB plates without any supplement (CK) or with NaCl (250, 500 and 750 mM), CuCl_2_ (250, 500 and 750 μM), CdCl_2_ (250, 500 and 750 μM) and ZnSO_4_ (250, 500 and 750 μM), respectively. NaCl, sodium chloride; CuCl_2_, copper chloride; CdCl_2_, cadmium chloride; ZnSO_4_, zinc sulfate.

As reported, plant chitinase was a stress-related gene, which could be induced by different stresses, such as mechanical wounding, ABA (abscisic acid), SA (salicylic acid), ZnSO_4_ (zinc sulfate), MeJA (methyl jasmonate) and so on [20,21]. Spot assay was performed to ascertain the response of Rosetta + pET 32a-*ScChi* and control (Rosetta + pET 32a) to different supplements *in vivo* (Figure 7). After overnight culture, Rosetta + pET 32a-*ScChi* showed faster growth as compared to that of the control on LB plates containing NaCl (sodium chloride), CuCl_2_ (copper chloride), CdCl_2_ (cadmium chloride) and ZnSO_4_. These results demonstrated that the recombinant protein could enhance the growth of recombinant cells in high stress condition.

### 2.6. Tissue-Specific Expression Analysis of *ScChi* in Different Sugarcane Tissues

**Figure 8.**
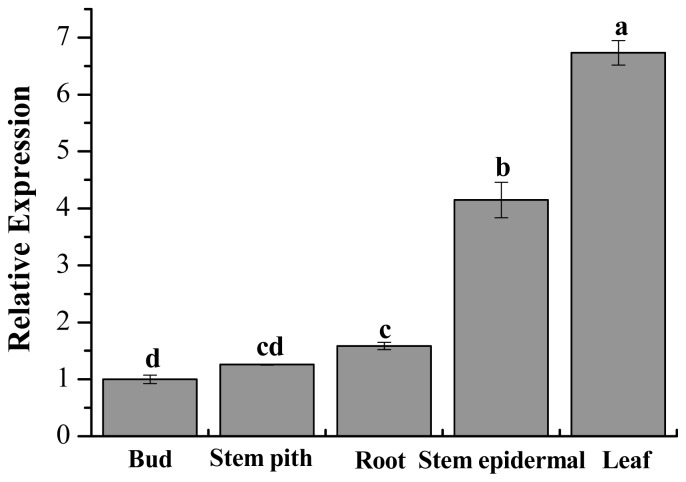
Tissue-specific expression analysis of the *ScChi* in different tissues of sugarcane cultivar Yacheng05–179. Data are normalized to the *GAPDH* expression level. All data points are the means ± SE (*n* = 3). Different lowercase letters indicate a significant difference, as determined by the least-significant difference test (*p*-value < 0.05).

For tissue-specific expression analysis of *ScChi*, the sugarcane cultivar, Yacheng05–179, was used as the experimental material. The expression of *ScChi* in root, leaf, bud, stem epidermal and stem pith was detected, with the *GAPDH* gene being used as an internal control for RT-qPCR. The results suggested that *ScChi* was highly expressed in leaf and stem epidermal (Figure 8), while the root and stem pith exhibited a moderate mRNA expression level, and the bud showed a relatively low level in comparison.

### 2.7. Expression Profiles of *ScChi* under Different Stress Treatments

**Figure 9.**
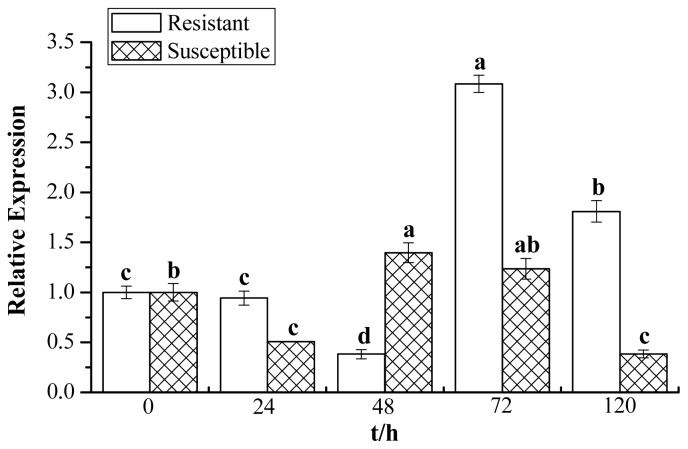
RT-qPCR (quantitative PCR) analysis of the *ScChi* expression patterns during sugarcane-smut (*Sporisorium scitamineum*) interaction. Data were normalized to the *GAPDH* expression level. All data points (with the deduction of their mocks) are the means ± SE (*n* = 3). Different lowercase letters indicate a significant difference, as determined by the least-significant difference test (*p*-value < 0.05). Resistant: Yacheng05–179 cultivar; susceptible: Liucheng03–182 cultivar.

Expression profiles of *ScChi* were studied by RT-qPCR after sugarcane (Yacheng05–179 and Liucheng03–182) was challenged with smut pathogen (Figure 9). The *ScChi* transcript was calculated by the expression level of the inoculated sample minus the level of the mock at each corresponding time point, so as to eliminate any effect of wounding. In response to smut stress, the transcript level of *ScChi* in the resistant cultivar of Yacheng05–179 exhibited a little decrease at 48 h, but showed increasing accumulation at 72 and 120 h, which was higher than that of *ScChi* in susceptible cultivar Liucheng03–182. During the interaction of Liucheng03–182 and smut, *ScChi* transcript decreased at 24 h, then elevated at 48 and 72 h and, again, decreased rapidly at 120 h. We could observed that the expression profiles of *ScChi* in the Yacheng05–179 and smut interaction were higher than those of *ScChi* in the Liucheng03–182 and smut interaction, except at 48 h.

**Figure 10.**
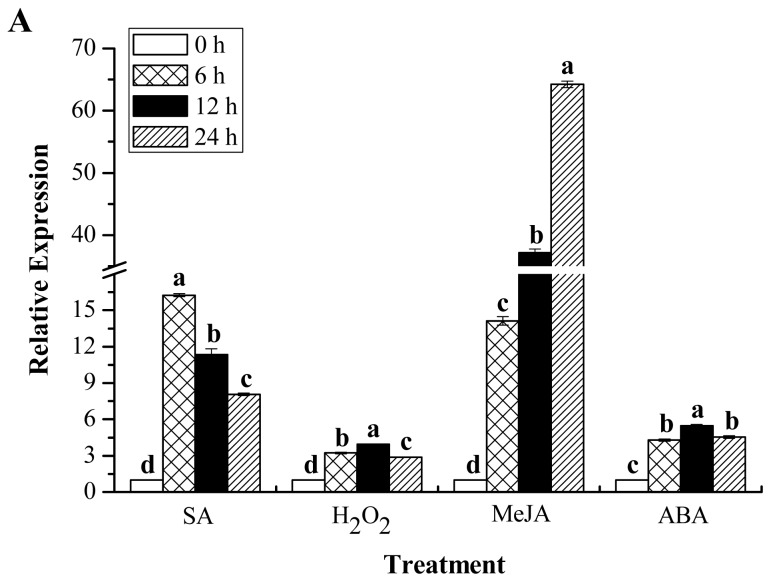

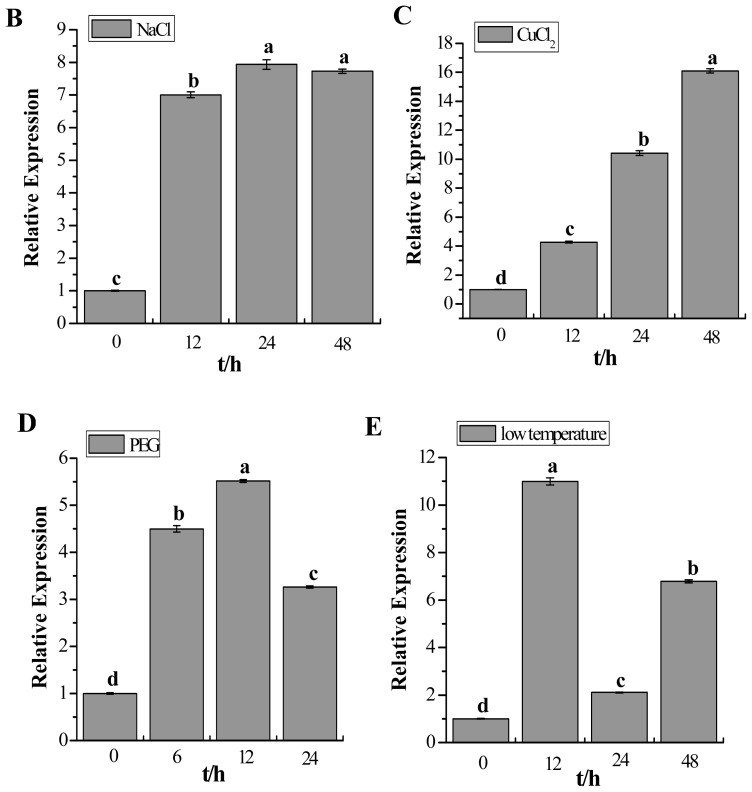
RT-qPCR analysis of the *ScChi* expression patterns in Yacheng05–179 plantlets under various abiotic stresses. Data were normalized to the *GAPDH* expression level. (**A**) The relative expression of *ScChi* under the stresses of 5 mM SA, 10 mM H_2_O_2_, 25 μM MeJA and 100 μM ABA; (**B**–**E**) the relative expression of *ScChi* under the stresses of 250 mM NaCl, 100 μM CuCl_2_, 25% PEG and 4 °C low temperature, respectively. All data points are the means ± SE (*n* = 3). Different lowercase letters indicate a significant difference, as determined by the least-significant difference test (*p*-value < 0.05). SA, salicylic acid; H_2_O_2_, hydrogen peroxide; MeJA, methyl jasmonate; ABA, abscisic acid; NaCl, sodium chloride; CuCl_2_, copper chloride; PEG, polyethylene glycol.

Furthermore, Yacheng05–179 plantlets under the stresses of 5 mM SA, 10 mM H_2_O_2_ (hydrogen peroxide), 25 μM MeJA, 100 μM ABA, 250 mM NaCl, 100 μM CuCl_2_, 25% PEG (polyethylene glycol) and 4 °C low temperature were carried out to examine the expression profiles of *ScChi* response to these stresses. As shown in Figure 10, the *ScChi* transcript was strongly induced by all of these exogenous stresses, including different plant hormones, oxidative stress, hyper-osmotic stress, metal stress, as well as temperature stress. It is interesting that under all the above abiotic treatments, *ScChi* showed a positive response in the early time of the stresses and maintained an increased transcript from 0 to 12 h or even to 24 h in the same cases of MeJA, NaCl and CuCl_2_ stresses.

### 2.8. Transient Expression of *ScChi* Induces a Defense Response in *N. benthamiana*

**Figure 11.**
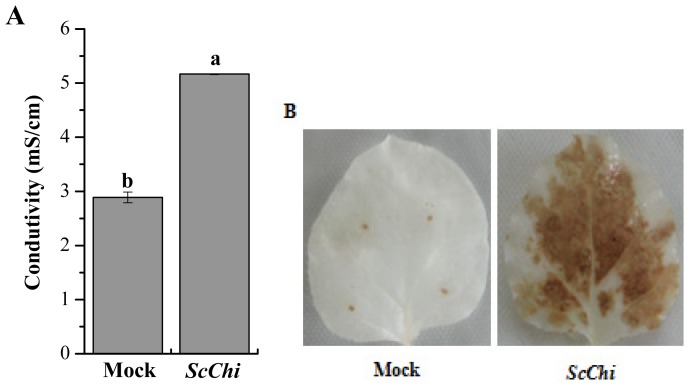
The transient expression of *ScChi* in *Nicotiana benthamiana* leaves. (**A**) Conductivity measurement of *N. Benthamiana* leaves infiltrated with the *35S::ScChi*-containing *Agrobacterium* strain after 48 h. Mock: the *Agrobacterium* strain carrying *35S::00*. All data points are the means ± SE (*n* = 3). Different lowercase letters indicate a significant difference, as determined by the least-significant difference test (*p*-value < 0.05); (**B**) DAB (3,3′-diaminobenzidinesolution) staining with *N. benthamiana* leaves 48 h after *Agrobacterium* strain infiltration.

**Figure 12.**
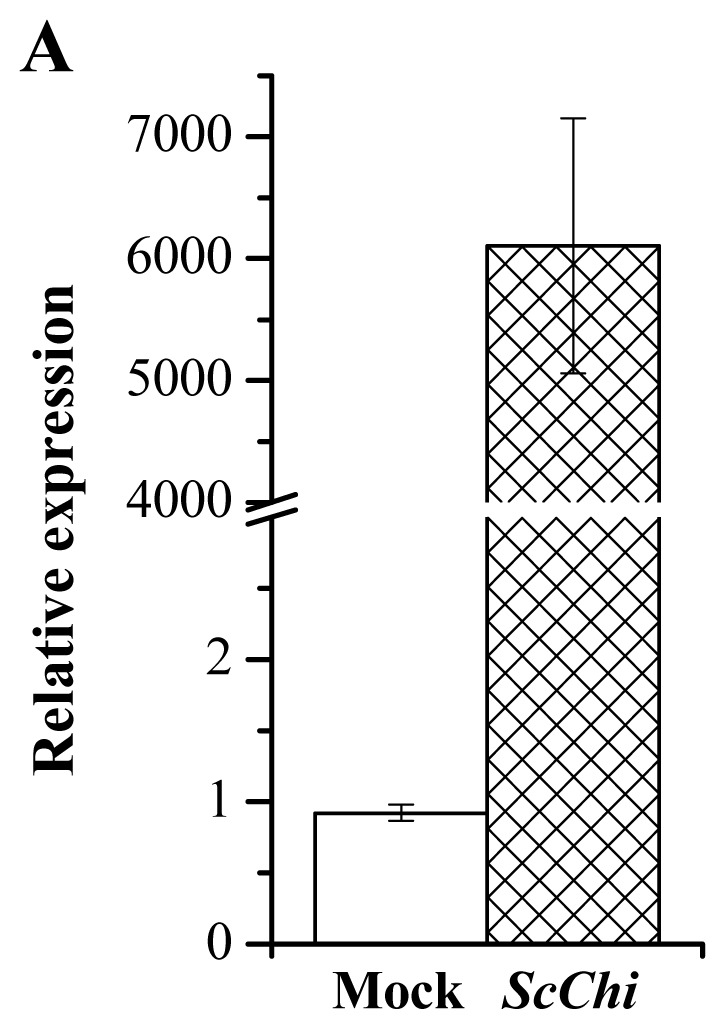

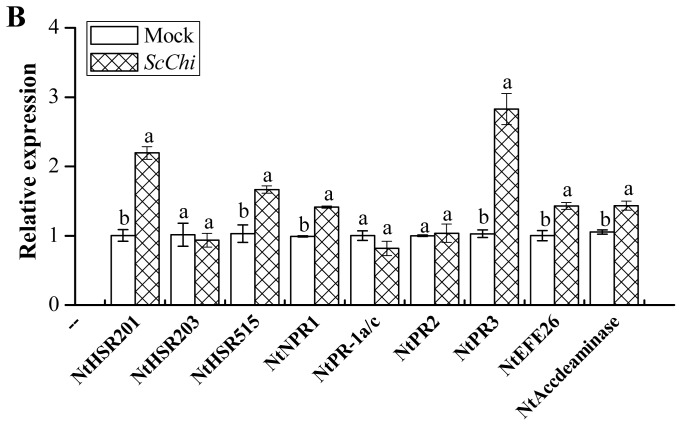
RT-qPCR analysis of *ScChi* and the immunity related marker genes in the *Nicotiana benthamiana* leaves 24 h after transiently expressing pCAMBIA 1301-*ScChi* and the empty vector. (**A**) The transcript analysis of *ScChi*; (**B**) the transcript analysis of the immunity related marker genes, including the hypersensitive response marker genes, *NtHSR201*, *NtHSR203* and *NtHSR515*, the salicylic acid related gene, *NtNPR1*, the jasmonate associated genes, *NtPR-1a/c*, *NtPR2* and *NtPR3*, and the ethylene synthesis depended genes, *NtEFE26* and *NtAccdeaminase. NtEF1-α* was used to normalize the transcript levels. Mock: the *Agrobacterium* strain carrying *35S::00*. All data points are the means ± SE (*n* = 3). Different lowercase letters indicate a significant difference, as determined by the least-significant difference test (*p*-value < 0.05).

The overexpressed vector of pCAMBIA 1301-*ScChi* was constructed and validated by the enzyme digesting identification. An *Agrobacterium*-mediated transient expression method was carried out to identify the effect of *ScChi* expression on the induction of the defense response in *N. benthamiana* leaves (Figure 11A,B). After 48 h, a typical hyper-sensitive response (HR) symptom with enhanced conductivity and deeper DAB (3,3′-diaminobenzidinesolution) staining color was found in the tested leaves expressing ScChi. On the contrary, plants transiently expressing the mock of pCAMBIA 1301 did not trigger defense response in *N. benthamiana* leaves. Moreover, the expression levels of *ScChi* and six immunity associated marker genes, including the HR marker genes, *NtHSR201* and *NtHSR515*, the SA-related gene, *NtNPR1*, the JA-associated gene, *NtPR3*, the ethylene-synthesis-depended genes *NtEFE26*, and *NtAccdeaminase*, were enhanced by the transient overexpression of *ScChi* (Figure 12).

## 3. Discussion

Plant diseases triggered by fungal pathogens are one of the major concerns in agriculture. Induced resistance to a plant pathogen is a complex mechanism, which involves the activation of various immune responses [1]. Many resistance genes, including PR proteins, have been isolated and used to enhance disease resistance in plants [22]. Initially, PR proteins were found commonly induced in resistant plants and present during HR to pathogens of bacteria, fungi and virus. However, it turned out that PR proteins can be triggered not only in resistant, but also in susceptible plants infected with pathogens, as well as in plants subjected to abiotic stresses [23,24]. Among these PR proteins, chitinases, which degrade the chitin walls of fungi, are widely distributed in nature and play a vital role in plant defense against pathogens. Hence, great effort has been made on the cloning and characterization of chitinase genes in plants [10]. Krishnaveni *et al.* have reported multiple antifungal chitinases, CH1, CH2 and CH3, from *Sorghum bicolor* [25]. Singh *et al.* found that a Class VII wheat chitinase exerted a broad-spectrum antifungal activity against *Sarocladium oryzae*, *Alternaria* sp., *C. falcatum*, *Rhizoctonia solani*, *Pestalotia theae* and *Fusarium* sp. [10].

Recently, Rahul *et al.* amplified several partial mRNA sequences of chitinase from both red rot-resistant and -susceptible sugarcane cultivars, and a full-length chitinase sequence, *ScChiB1*, was isolated from the resistant cultivar [18]. An upregulated (2.43-fold) expression protein of chitinase was identified in the leaves of chewing cane inoculated with *C. falcatum*, and the activity of chitinase was enhanced and differed significantly in the 10 chewing cane cultivars [19]. In addition, Viswanathan *et al.* performed a Western blotting study and found a higher and rapid accumulation of chitinase in a red rot-resistant cultivar [26]. The present study revealed that chitinase was also triggered during the interaction between sugarcane and *S. scitamineum*. What should also be stressed is that the chitinase enzyme activity in the resistant cultivar (Yacheng05–179) was almost always higher than those of the mock and the susceptible cultivar (Liucheng03–182) (Figure 1), suggesting that this chitinase may be involved in the induction of sugarcane resistance against *S. scitamineum*.

Chitinases have been reported to group into seven classes (Class I–VII) belonging to the glycoside hydrolase families 18 and 19, suggesting that chitinase isozymes were encoded by a multi-gene family [2,10]. In the present study, we isolated a chitinase gene that was induced upon infection of *S. scitamineum* at the time point of 48 h. Multiple sequence alignment showed 84.27% identity of ScChi (KF664180) with *Z. mays* chitinase (AFW72831.1), 75.78% with that of *A. tauschii* (EMT04102.1), 73.61% with that of *O. sativa* (NP_001064607.1) and 73.45% with that of *T. urartu* (EMS51632.1). The deduced amino acid sequences showed that there was an active site of the catalytic residues, Asp_137_ (D_137_), Asp_140_ (D_140_), Asp_142_ (D_142_) and Glu_146_ (E_146_) (DXXDXDXXXE), of glycosyl hydrolase family 18 in ScChi. Park *et al.* have reported that substitution at any of these sites (D or E) could result in the reduction or elimination of chitinase activity [27]. The obtained dendrogram in this study revealed that ScChi was closely related to *O. sativa* Class III chitinase (AAK26395) (Figure 3). Therefore, ScChi may be suggested for catalyzing the chitinase activity as an acid form of Class III (belonging to glycosyl hydrolase family 18). Using the *Agrobacterium*-mediated transformation in *N. benthamiana* leaves, the ScChi::GFP fusion protein was observed in the nucleus, cytoplasm and the plasma membrane (Figure 5). Results also revealed that ScChi, which contained no signal peptide and transmembrane helix domain, was unlikely to be a secretory protein. Previous studies even revealed extracellular localization of acidic Class III chitinases from chickpea (*Cicer arietinum*) and *N. tabacum* [3,28].

Chitinases are induced systematically on biotic, as well as abiotic treatments [1,29]. In the present study, the recombinant protein, ScChi, showed better growth in *E. coli* cells under all the four kinds of abiotic stress conditions, the NaCl, CuCl_2_, CdCl_2_ and ZnSO_4_ treatments. Previous studies have also demonstrated better growth of *E. coli* under stresses for recombinant protein production [30,31]. Chaurasia *et al.* found that phytochelatin synthase gene, *PCS*, was better expressed in *E. coli* by the addition of salt, pesticide, cadmium, copper, heat and UV [32]. The recombinant protein of sugarcane dirigent protein gene, *ScDir*, could tolerate high PEG and NaCl stresses [31]. Here, *ScChi* showed an upregulated transcript after challenge with smut pathogen. It also revealed that the expression level of *ScChi* in the incompatible interaction (Yacheng05–179 *vs.* smut pathogen) remained higher than that in a compatible interaction (Liucheng03–182 *vs.* smut pathogen) (Figure 9). Interestingly, the *ScChi* transcript was strongly induced by hormones stresses (SA, MeJA and ABA), especially by SA and MeJA, oxidative stress (H_2_O_2_), hyper-osmotic stresses (NaCl and PEG), metal stress (CuCl_2_), as well as low temperature (4 °C) stress (Figure 10). Li *et al.* found that the expression level of chitinase gene *Mmchi1* in *Mikania micrantha* was significantly upregulated after two days post infection by *Cuscuta campestris* and also increased in response to the stresses of SA, ABA, ZnSO_4_ and wounding [20]. An acidic Class III chitinase, suggested to be a pathogenesis protein, was purified from *N. tabacum* leaves infected with *Tobacco mosaic virus* (TMV) [3]. Rahul *et al.* obtained a Class IV chitinase gene with a high level of expression observed in the incompatible interaction during challenge with *C. falcatum* causing red rot, although this gene was expressed in both resistant and susceptible sugarcane cultivars [18]. These phenomena suggest that the regulatory mechanism of chitinase genes might be similar in both eukaryotes and prokaryotes under biotic/abiotic stimuli. In the present study, enhanced chitinase activity and the expression profiles of the sugarcane chitinase gene, *ScChi*, during various environmental stresses suggest the positive responses to biotic and abiotic stresses.

It has been reported that many chitinase genes are developmentally regulated and may play a part in the specific physiological processes [33]. In our study, the transcript of *ScChi* accumulated in different sugarcane tissues (Figure 8). Constitutive expression of *ScChi* was detected, high in leaf and stem epidermal, moderate in root and stem pith and low in bud, indicating a specific role of *ScChi* in leaf and stem epidermal tissues. Infection with an avirulent pathogen strain or microbial triggering caused the rapid production of reactive oxygen intermediates (ROI) [34]. During the expression of plant disease resistance, H_2_O_2_ played an important role in the orchestration of a localized hypersensitive response [34]. In the present study, as indicated in Figure 11, pCAMBIA 1301-*ScChi* overexpressed in *N. benthamiana* leaves exhibited deep DAB staining color and high conductivity, indicating the high level of H_2_O_2_ accumulation. Additionally, the expression levels of *ScChi* and six immunity associated marker genes, *NtHSR201*, *NtHSR515*, *NtNPR1*, *NtPR3*, *NtEFE26* and *NtAccdeaminase*, were upregulated (Figure 12). These results suggest a close relationship between the expression of *ScChi* and plant immunity, which was consistent with previous reports [34].

## 4. Experimental Section

### 4.1. Plant Materials and Treatments

Sugarcane cultivars of Yacheng05–179 (resistant) and Liucheng03–182 (susceptible) were cultivated in the Key Laboratory of Sugarcane Biology and Genetic Breeding, Ministry of Agriculture (Fuzhou, China). Smut whips were also collected from sugarcane cultivar “ROC”22, then sealed in a plastic bag and stored at 4 °C. The germination rate of the spores was checked before inoculation. A bath of two-bud sets of both sugarcane cultivars, Yacheng05–179 (smut resistant) and Liucheng03–182 (smut susceptible) (private bulletin), were inoculated with 0.5 μL suspension, which contained 5 × 10^6^ spores/mL in 0.01% (*v*/*v*) Tween-20 [35]. As a control, a bath of two-bud sets were mock-inoculated with 0.01% (*v*/*v*) Tween-20 in sterile distilled water [36,37]. The treated samples were maintained at 28 °C in a photoperiod of 12 h light/12 h dark. Five buds were excised at 0, 24, 48, 72 and 120 h post-infection and stored at −80 °C, respectively.

To investigate gene expression in different tissues, six uniform 10-month-old plants of Yacheng05–179 were chosen for analysis. For each plant, the youngest fully expanded leaf (+1 leaf) with a visible dewlap (the collar between the leaf blade and sheath), the young root, the stem epidermal, stem pith and all the buds were collected and fixed in liquid nitrogen before RNA extraction.

For abiotic treatments, 4-month-old sugarcane tissue culture plantlets of Yacheng05–179 were subjected to the following eight different treatments in conical tubes at 28 °C with 16 h light/8 h dark. The plantlets were treated with 5 mM SA solution, 25 μM MeJA in 0.1% (*v*/*v*) ethanol and 0.05% (*v*/*v*) Tween-20, 100 μM ABA and 25% PEG, respectively. For the above four treatments, the whole plantlets were harvested at 0, 6, 12 and 24 h, respectively. In addition, the whole plantlets were separately treated with 250 mM NaCl, 100 μM CuCl_2_ and 4 °C low temperature for 0, 12, 24 and 48 h [37,38]. For H_2_O_2_ stress, leaves were sprayed with 10 mM H_2_O_2_, and the whole plantlets were sampled at 0, 6, 12 and 24 h [39]. All of the treatments were carried out in three replicates.

### 4.2. Chitinase Activity Assay

Chitinase activity was tested by measuring the reducing end group *N*-acetamino-glucose produced from colloidal chitin [25,40]. Yacheng05–179 and Liucheng03–182 inoculated with smut pathogen, as well as mock controls (0, 24, 48, 72 and 120 h) were sampled as above. Buds of 0.5 g were homogenized with 3.0 mL of ice-cold sodium acetate buffer (pH 5.0), then centrifuged at 10,000× *g* for 10 min at 4 °C. Additionally, the supernatant was recentrifuged again in the same condition. The final supernatant was used as a crude enzyme solution. The reaction mixture (1.0 mL enzyme solution (for blank control, incubated in boiling water for 10 min), 2.0 mL of 0.1 M sodium acetate buffer (pH5.0) and 0.1 g colloidal chitin) was incubated at 37 °C overnight. Termination of the reaction was done by heating in boiling water for 10 min. The reaction was then cooled to room temperature and centrifuged at 4000 r/min for 5 min. The supernatant of 2.0 mL was mixed with 2.0 mL dinitrosalicylic acid reagent and subjected to boiling water for 10 min. After that, 6.0 mL distilled water was added, and spectrophotometric measurement by the Lambda 35 UV WinLab software (Perkin Elmer, New York, NY, USA) at 540 nm was conducted. One unit of chitinase activity (U) was defined as the amount of enzyme that liberates 1.0 μmol *N*-acetamino-glucose per gram of plant material. The activity of chitinase was calculated by the activity level of inoculation minus the level of the mock at each corresponding time point.

### 4.3. RNA Extraction and cDNA Synthesis

Total RNA was extracted using Trizol reagent (Invitrogen, Carlsbad CA, USA), according to the manufacturer’s recommendations. RNA quality was determined by electrophoresis and spectrophotometer (NanoVue plus, GE, Schenectady, New York, NY, USA). DNase I (Promega, Madison, WI, USA) treated total RNA was used for first-strand cDNA synthesis by the Prime-Script™ RT Reagent Kit (Takala, Dalian, China).

### 4.4. Cloning, Sequencing and Bioinformatic Analysis

Twenty-two sugarcane expressed sequence tags (ESTs) were obtained from the sugarcane sequence database (cultivated sugarcanes (taxid: 286192); wild sugarcane (taxid: 62335); sugarcane (taxid: 128810); sugarcane (taxid: 4547)) in GenBank, which share high homology with the *Z. mays* chitinase mRNA sequence (GenBank accession no. NM_001154758.1). A putative cDNA sequence of sugarcane chitinase gene was assembled by the CAP3 sequence assembly program [41]. PCR was performed with primers ScChi-cDNAF and ScChi-cDNAR (Table 1) derived from the assembled sequence, using the Yacheng05–179 post-48 h *S. scitamineum* inoculation cDNA sample as a template. The PCR products were gel-purified, cloned into the pMD18-T vector (TaKaLa, Dalian, China) and sequenced (Shenggong, Shanghai, China).

The open reading frame (ORF) was analyzed using the ORF Finder online program [42]; ProtParam [43], SMART [44], NetNGlyc 1.0 Server [45], SignalP 4.0 Server [46], TargetP 1.1 server [47] and TMHMM Server v. 2.0 [48] were used to predicted the *ScChi* sequence. The ClustalW method available in the MEGA5.05 software [49] was employed for multiple sequence alignment of amino acid sequences of *ScChi* with other published plant chitinases in GenBank [10]. The phylogenetic tree was constructed by the neighbor-joining (NJ) method (1,000 bootstrap replicates) with the MEGA 5.05 program [50].

### 4.5. Subcellular Localization Assay

*ScChi* was PCR amplified from pMD18-T-*ScChi* using primers ScChi-SublocF and ScChi-SublocR (Table 1). Then, the fragment was fused with the pCAMBIA 2300-GFP constructor between the *Xba* I and *Spe* I sites to generate the subcellular location vector of *35S::ScChi::GFP*. The recombinant vector was verified by PCR, double digestion and sequencing. A positive clone was transferred into *Agrobacterium tumefaciens* strain EHA105. *N. benthamiana* leaves at eight-leaf stage were chosen for the *Agrobacterium*-mediated transformation assay [51,52]. *Agrobacterium* cells containing the *35S::GFP* or *35S::ScChi::GFP* vector were grown overnight at 28 °C in LB liquid medium (containing 50 μg/mL kanamycin and 35 μg/mL rifampicin). The *Agrobacterium* solution (OD_600_ = 0.8) consisting of 200 μM acetosyringone was infiltrated into *N. benthamiana* leaves and cultured for 2 days (16 h light/8 h darkness) at 24 °C. The subcellular localization of the fusion protein was analyzed by fluorescence microscopy (Axio Scope A1, Heidenheim, Germany).

### 4.6. Expression in *E. coli* Rosetta

The ORF of *ScChi* was amplified by PCR from pMD18-T-*ScChi*, using the primers, ScChi-32aF and ScChi-32aR (Table 1), followed by initial denaturation for 4 min at 94 °C; 35 cycles of 30 s at 94 °C, 30 s at 58 °C and 1.5 min at 72 °C; and extension for 10 min at 72 °C. The PCR fragments were digested by the *Eco*RI and *Xho*I enzymes and ligated into the previously digested pET 32a (+) vector. The ligation mixture was used to transform *E. coli* Rosetta competent cells to construct the recombinants. The construct was verified by PCR amplification, double digestion and sequencing, and the positive clone was named pET 32a-*ScChi*. The prokaryotic expression of the recombinant protein was induced by the addition of 1.0 mM isopropyl β-d-thiogalactoside (IPTG) for 1 h at 28 °C. LB medium with blank *E. coli* Rosetta (blank) or Rosetta + pET 32a (control) was also induced in IPTG for 1 h. All the above prokaryotic expression samples were analyzed by sodium dodecyl sulfate-polyacrylamide gel electrophoresis (SDS-PAGE).

For the study of the stress responses of *E. coli* cells expressing the *ScChi* gene under different abiotic conditions, a spot assay in treatments of NaCl, CuCl_2_, CdCl_2_ or ZnSO_4_ was carried out. When OD_600_ of the LB medium (with 80 μg/mL ampicillin and 170 μg/mL chloramphenicol) with *E. coli* cells plus pET 32a-*ScChi* or pET 32a reached 0.6, 1.0 mM IPTG was added, and the cells were grown for 12 h at 37 °C. The cultures were firstly diluted to 0.6 at OD_600_ and, then, diluted to two levels (10^−3^ and 10^−4^). Ten microliters from each dilution were spotted on LB plates with 80 μg/mL ampicillin and 170 μg/mL chloramphenicol or containing NaCl (250, 500 and 750 mM), CuCl_2_ (250, 500 and 750 μM), CdCl_2_ (250, 500 and 750 μM) or ZnSO_4_ (250, 500 and 750 μM). The tested plates were cultured at 37 °C overnight and photographed.

### 4.7. Expression Patterns of *ScChi* in Different Tissues and Stress Treatments

Expression profiles of *ScChi* during Yacheng05–179-smut interaction and Liucheng03–182-smut interaction at 0, 24, 48, 72 and 120 h, as well as mock plants were investigated. The *ScChi* transcript under *S. scitamineum* stress was calculated by the expression level of the inoculated sample minus the level of the mock at each corresponding time point. Expression patterns of *ScChi* in different tissues (leaf, root, bud, stem epidermal and stem pith) and in different stress treatments (SA, H_2_O_2_, MeJA, ABA, NaCl, CuCl_2_, PEG and 4 °C low temperature) were performed by RT-qPCR.

RT-qPCR was carried out with the SYBR Green Master (ROX) kit (Roche, Shanghai, China) on a 7500 RT-qPCR system (Applied Biosystems, Foster, CA, USA). The primer sequences for *ScChi* (ScChi-QF/ScChi-QR) and *GAPDH* (GAPDH-QF/GAPDH-QR) [53] were shown in Table 1. The RT-qPCR was performed in a final volume of 20 μL, which contained 0.5 μM of each primer, 10 μL FastStart Universal SYBR Green PCR Master (ROX) (Roche, Shanghai, China) and 2.0 μL template (10× diluted cDNA). PCR with distilled water as the template was performed as the blank control. The RT-qPCR cycling conditions were as follows: 50 °C for 2 min, 95 °C for 10 min, 40 cycles of 95 °C for 15 s and 60 °C for 1 min; and the melting curve was established at the end of the amplification. Each RT-qPCR was conducted in triplicate. The 2^−Δ Δ^*^C^*^t^ method was used to calculate the relative gene expression [54].

### 4.8. Transient Expression of *ScChi* in *N. benthamiana*

The primers of ScChi-1301F/ScChi-1301R in Table 1 were used to construct the overexpression vector pCAMBIA 1301-*ScChi* to analyze its defense response. *Agrobacterium* strain EHA105 carrying the recombinant vector was grown overnight in LB liquid medium containing 50 μg/mL kanamycin and 35 μg/mL rifampicin at 28 °C. Culture cells were collected and resuspended in MS liquid medium containing 200 μM acetosyringone at OD_600_ = 0.8. Then, cells were infiltrated into eight-leaf stage-old *N. benthamiana* leaves [51,52]. For comparison, the *Agrobacterium* strain containing the pCAMBIA 1301 vector alone was also transiently expressed in *N. benthamiana* leaves. One of the materials were incubated at 24 °C for 24 h (16 h light/8 h darkness) and used for the RT-qPCR analysis of the expression of *ScChi* (ScChi-QF/ScChi-QR) and several immunity associated marker genes, including the hypersensitive response (HR) marker genes, *NtHSR201*, *NtHSR203* and *NtHSR515*, the SA-related gene, *NtNPR1*, the JA-associated genes, *NtPR-1a/c*, *NtPR2* and *NtPR3*, and the ethylene synthesis-dependent genes, *NtEFE26* and *NtAccdeaminase* (Table 1), in *N. benthamiana. NtEF1-α* (Table 1) was used to normalize the transcript levels. Other materials were incubated at 24 °C for 2 days and applied to the following tests of the histochemical assay and ion conductivity determination. All of the treatments were carried out in three replicates.

### 4.9. Histochemical Assay

DAB (3,3′-diaminobenzidinesolution) was used to stain H_2_O_2_ produced in *N. benthamiana* leaves. The leaves were incubated in 1.0 mg/mL DAB-HCl solution in the dark overnight and destained by boiling in 95% ethanol for 5 min. The bronzing color of the leaves for H_2_O_2_ detection was photographed [52,55].

### 4.10. Measurement of Ion Conductivity

Ion conductivity determination was performed as in the research of Hwang and Hwang [51]. Six leaf discs (11 mm in diameter) per leaf were washed and incubated in 20 mL of distilled water and then shaken gently for 60 min at room temperature. The ion conductivity was recorded using a conductivity meter (SevenEasy, METTLER TOLEDO, Zurich, Switzerland).

## 5. Conclusions

In summary, this is a report of the activity and cloning of sugarcane chitinases involved in the defense response to *S. scitamineum*. The pathogenesis-related induction of a 31.37 kDa chitinase, ScChi, was clearly identified at the transcript and protein levels. *ScChi* showed a positive response to biotic and abiotic stresses. Although the defensive role of chitinase in sugarcane against fungi is far from being fully understood, our study is indicative of the involvement of chitinase in the resistance response of sugarcane to biotic and various abiotic stimuli. Whether chitinase induction plays a direct role in the resistance of sugarcane to *S. scitamineum* needs further study. The results obtained in this study can be used for smut management in sugarcane and the development of marker-assisted breeding if the positive response to smut challenge can be found in sugarcane from different genetic backgrounds.

## Figures and Tables

**Table 1.** Primers used in this study.

Primer	Sequence (5′-3′)	Strategy
ScChi-cDNAF	ATACAGGTCCATTTTGTCAGC	RT-PCR
ScChi-cDNAR	AAACCAAGCAAGCCCTAA	RT-PCR
ScChi-SublocF	TGCTCTAGAATGACGAACGGCTACC	Subcellular localization vector construction
ScChi-SublocR	GGACTAGTGTGGTTGGCAACGATCT	Subcellular localization vector construction
ScChi-32aF	CGGGATCCATGACGAACGGCTACCT	prokaryotic expression vector construction
ScChi-32aR	CCAAGCTTTCAGTGGTTGGCAACGA	prokaryotic expression vector construction
ScChi-QF	ACGGCTACGGCGACAACA	RT-qPCR
ScChi-QR	GTCCGCTGACCAGATGAAGAG	RT-qPCR
GAPDH-QF	CACGGCCACTGGAAGCA	RT-qPCR
GAPDH-QR	TCCTCAGGGTTCCTGATGCC	RT-qPCR
ScChi-1301F	TGCTCTAGAATGACGAACGGCTACCTG	Over expression vector construction
ScChi-1301R	CGGGATCCTCAGTGGTTGGCAA	Over expression vector construction
NtHSR201 F	CAGCAGTCCTTTGGCGTTGTC	RT-qPCR
NtHSR201 R	GCTCAGTTTAGCCGCAGTTGTG	RT-qPCR
NtHSR203 F	TGGCTCAACGATTACGCA	RT-qPCR
NtHSR203 R	GCACGAAACCTGGATGG	RT-qPCR
NtHSR51 F	TTGGGCAGAATAGATGGGTA	RT-qPCR
NtHSR51 R	TTTGGTGAAAGTCTTGGCTC	RT-qPCR
NtNPR1 F	GGCGAGGAGTCCGTTCTTTAA	RT-qPCR
NtNPR1 R	TCAACCAGGAATGCCACAGC	RT-qPCR
NtPR-1a/c F	AACCTTTGACCTGGGACGAC	RT-qPCR
NtPR-1a/c R	GCACATCCAACACGAACCGA	RT-qPCR
NtPR2 F	TGATGCCCTTTTGGATTCTATG	RT-qPCR
NtPR2 R	AGTTCCTGCCCCGCTTT	RT-qPCR
NtPR3 F	CAGGAGGGTATTGCTTTGTTAGG	RT-qPCR
NtPR3 R	CGTGGGAAGATGGCTTGTTGTC	RT-qPCR
NtEFE26 F	CGGACGCTGGTGGCATAAT	RT-qPCR
NtEFE26 R	CAACAAGAGCTGGTGCTGGATA	RT-qPCR
NtAccdeaminase F	TCTGAGGTTACTGATTTGGATTGG	RT-qPCR
NtAccdeaminase R	TGGACATGGTGGATAGTTGCT	RT-qPCR
NtEF1-α F	TGCTGCTGTAACAAGATGGATGC	RT-qPCR
NtEF1-α R	GAGATGGGGACAAAGGGGATT	RT-qPCR
